# The Application of Next-Generation Sequencing (NGS) in Neonatal-Onset Urea Cycle Disorders (UCDs): Clinical Course, Metabolomic Profiling, and Genetic Findings in Nine Chinese Hyperammonemia Patients

**DOI:** 10.1155/2020/5690915

**Published:** 2020-08-31

**Authors:** Qingnv Zhou, Huafei Huang, Li Ma, Tianwen Zhu

**Affiliations:** ^1^Department of Neonatology, Jiaxing Maternity and Child Health Care Hospital, Jiaxing, China; ^2^Department of Neonatology, Shanghai Children's Hospital, Shanghai Jiao Tong University School of Medicine, Shanghai, China; ^3^Department of Neonatology, Xinhua Hospital, Shanghai Jiao Tong University School of Medicine, Shanghai, China

## Abstract

During Jan. 2016–Dec. 2019, nine Chinese patients from eight unrelated families were diagnosed with neonatal-onset UCDs by targeted panel sequencing or whole-exome sequencing (WES). Their clinical manifestations, biochemical features, 180-day-age outcomes, and molecular genetic characteristics were reviewed retrospectively. NGS-based tests revealed 7 patients diagnosed with ornithine transcarbamylase deficiency (OTCD) and 2 with carbamoylphosphate synthetase I deficiency (CPS1D). The spectrum of the clinical presentation of nine affected individuals progressed from unspecific symptoms like poor feeding to somnolence, coma, and death. All patients presented with an acute hyperammonemia. The most robust metabolic pattern in OTCD was hyperglutaminemic hyperammonemia with high concentration of urine orotic acid, and it was reported in six patients. Of ten variants found on the *OTC* gene and CPS1 gene, 3 were novel: (c.176T>C (p.L59P)) in the *OTC* gene, c.2938G>A (p.G980S) and c.3734T>A (p.L1245H) in the *CPS1* gene. There was a high mortality rate of 77.78% (7/9) for all the defects combined. An OTC-deficient male and a CPS1-deficient female survived from episodes of hyperammonemia. Although prompt recognition of UCD and the use of alternative pathway therapy in addition to provision of appropriate nutrition and dialysis improved survival, the overall outcomes for the neonatal-onset type are poor in China.

## 1. Introduction

Neonatal-onset UCDs [1] are inborn errors of metabolism characterized by episodic, life-threatening hyperammonemia within the first 28 days of life. The severity of early-onset UCDs depends on the specific variants involved and their impacts on the corresponding enzymatic or transport function. CPS1D [2] is the most severe of the UCDs, and individuals with complete inactivity of CPS1 rapidly develop hyperammonemia after birth. Absence of OTC activity [3] in males is as severe as CPS1D. However, the acute metabolic crises in early life may be misdiagnosed, as the clinical features are nonspecific and mimic infections. Therefore, it was imperative to emphasize neonatal-onset UCDs, where timely identification and immediate intervention is of key importance for the outcomes of the patients.

The diagnosis of UCDs is generally established by blood tandem mass spectrometry (MS/MS), urine gas chromatography mass spectrometry (GC/MS) [4], and molecular analysis [5]. The latter is the primary method of diagnostic confirmation for all UCDs. Today, different NGS techniques can be used for diagnostic purposes, and the value of NGS diagnostics in the intensive care setting is undisputed [6].

The overall prevalence of UCDs has recently been determined to be 1 in 35,000 births [7], although it varies between populations. With improvements in health care, the mortality in neonatal-onset cases has significantly decreased from previous reports of 50% [8] to 24% [7] nowadays. To date, there have been several studies of UCDs in China [9–11]. However, few focus on the relation between the clinical course, metabolomic profiling, molecular genetic of severe neonatal-onset types, and their survivals.

The primary purpose of the study was to summarize the clinical, biochemical, and genetic features and 180-day-age outcome of nine Chinese patients with neonatal-onset UCDs detected by NGS-based tests retrospectively.

## 2. Materials and Methods

### 2.1. Patients and Clinical Data

This is a retrospective study of clinical course, metabolomic profiling, and genetic findings for 9 patients diagnosed as having a UCD at the corresponding neonatal intensive care units (NICUs) from three tertiary hospitals in China. The institutions included Xinhua Hospital affiliated with Shanghai Jiao Tong University School of Medicine (XH), Children's Hospital affiliated with Shanghai Jiao Tong University (CH), and Jiaxing Maternity and Child Health Care Hospital (JX). The analysis and publication of data related to the study were approved by the institutional review board at Xinhua Hospital, Shanghai Jiao Tong University School of Medicine (Approval number: XHEC-D-2019-101) with a waiver of consent and authorization.

The diagnosis of UCDs was based on the biochemical findings or molecular analyses of the genes involved in the urea cycle pathway. The phenotypes of the affected neonates were further translated into Human Phenotype Ontology (HPO) [12] terms. All patients were followed up till six months after birth. The primary end point was survival of the episode of hyperammonemia during the follow-up interval. When a patient died, the investigator was asked to identify the primary and secondary causes of death and to assess the relationship of death to the primary disease.

Data regarding gestational age, birth weight, family history, and other demographic, clinical, biochemical, and related molecular results were extracted from the medical records of the patients. Suspicion of genetic causes was based on clinical evaluation by experienced neonatologists.

### 2.2. Advanced Diagnostic Biochemical Investigations

Besides the blood ammonia, advanced biochemical investigations carried out for diagnosis of UCDs included blood mass spectrometry profile by MS/MS (API 4000, American Bio-Systems Inc.) using blood filter papers and urine organic acid analysis by GC-MS (Shimadzu Limited, QP2010).

### 2.3. Genetic Sequencing

Genomic DNA was extracted from the peripheral whole blood of patients and their parents using a QIAamp Blood DNA Mini Kit (Qiagen GMBH, Hilden, Germany). Four out of five patients from XH underwent targeted exome sequencing (TES) with a specific neonatal metabolism disease 175-gene panel (MyGenostics) (see Supplementary Method for details), and the rest underwent whole-exome sequencing (WES). For WES, the capture probes were those used in GenCap Custom Exome Enrichment Kits (MyGenostics, Beijing, China) and TruSight Rapid Capture Kits (Illumina, Inc., San Diego, CA, USA). Captured libraries were sequenced by an Illumina HiSeq 6000. The Burrows-Wheeler Aligner (BWA, version 0.7.10) was used to align the reads to the human reference genome (GRCh37/hg19). Copy number variations (CNVs) and small variants were identified using VarScan 2 and Genome Analysis Toolkit (GATK) (4.0.10.1).

### 2.4. Data Analysis

Detected variants were interpreted and categorized according to the five-tier classification system recommended by the American College of Medical Genetics [13, 14]. In addition, they were addressed as novel or reported according to entries into the Human Gene Mutation Database (http://www.hgmd.org) [15] and the Genome Aggregation Database (gnomAD; http://gnomad.broadinstitute.org) [16]. Deleterious mutations and novel variants detected by NGS were confirmed via Sanger sequencing. The following, available online analysis tools were used to predict the effect of the novel missense variants. PolyPhen-2 [17] and sorting intolerant from tolerant (SIFT) [18] were used to predict their pathogenic effects. The multiple-sequence alignments were carried out by ClustalX [19]. Protein structure analysis of missense variants were performed by Project HOPE [20]. The human OTC (UniProtKB/Swiss-Prot P00480) and CPS1 (UniProtKB/Swiss-Prot P31327) protein sequences were used as the reference sequence. Potential CNVs, if any, identified by WES [21] were further examined by karyotype testing or chromosomal microarray analysis (CMA).

Patients were considered to have a laboratory-confirmed genetic diagnosis if they had a pathogenic variant or likely pathogenic variant detected by a genetic test that explained the patient's clinical presentation. For CPS1D patients with one variant of uncertain significance (VUS) [22] combined with a pathogenic or likely pathogenic variant, the VUS was also considered to be disease-causing if the phenotype appropriately matched.

## 3. Results

### 3.1. Clinical Features of Patients with Neonatal-Onset UCDs

Table 1 shows the demographic and clinical findings on nine patients with neonatal-onset UCDs from 8 unrelated families. The patient cohort consisted of 7 males with OTCD (P1, P2, P3, P4, P5, P6, and P9) and 2 with CPS1D (P7 and P8). Three patients (P5, P6, and P9) were premature neonates (<37 weeks gestation) with a low birth weight (<2500 g); two of them were siblings (P5 and P6). All patients presented acute encephalopathy with different degrees at the age of 3.78 ± 2.49 days (range, 0–7 days): from somnolence on one extreme of the symptom spectrum to coma on the other. High 180-day mortality (77.78%, 7/9) was observed in this cohort.

Five patients (P3, P5, P6, P8, and P9) appeared normal at birth but progressed to seizures or coma within one to three days of age in hospitals. In particular, one preterm boy (P9) presented with tachypnea one hour after birth, deteriorated rapidly, and died at 3 days of age. His mother was a heterozygote for the *OTC* gene and developed acute hyperammonemia during pregnancy. The remaining four patients (P1, P2, P4, and P7) were discharged from their delivery hospitals within three to five days after birth. Their initial symptoms were nonspecific, such as somnolence or hypothermia or poor feeding, which developed at home and resembled a neonatal infection.

### 3.2. Metabolomic Profiling of Patients with Neonatal-Onset UCDs

The biochemical analysis of each patient is summarized in Table 1. An episode was defined as a single hospitalization for hyperammonemia. We referred the first known hyperammonemia episode of the patient as the first hospitalization for his/her acute hyperammonemia, and we selected the pretreatment peak ammonium level for the first episode.

A total of 10 episodes of acute hyperammonemia were derived from our study. Eight patients (P1, P3, P4, P5, P6, P7, P8, and P9) had one hyperammonemia episode, whereas a male OTCD (P2) had two before 30 days of age. Plasma ammonia level varied among our patients, with the mean value of 696.10 ± 424.59.07 *μ*mol/L (range, 228.60-1367.00 *μ*mol/L) (reference interval: 9-30 *μ*mol/L). The presence of hyperlactatemia and metabolic acidosis was found in seven (P2, P3, P4, P5, P6, P8, and P9) and four patients (P2, P5, P6, and P9), respectively. Only one CPS1-deficient male (P8) developed a severe respiratory alkalosis at admission.

Plasma amino acid and urine organic acid analyses were performed in all these patients. Metabolomic finding of OTCD subjects (Table 1) revealed high serum glutamine associated with high urine orotic acid in six cases (P2, P3, P4, P5, P6, and P9) and low plasma citrulline in four (P4, P5, P6, and P8). Fewer metabolite perturbations were identified in CPS1D patients (Table 1). We found low plasma citrulline (1.58 *μ*mol/L, reference interval 5-40 *μ*mol/L) and hyperglutaminemia (1104.41 *μ*mol/L, reference interval 2-35 *μ*mol/L) in one patient (P8), while their plasma arginine and urinary orotic acid were normal (P7 and P8).

### 3.3. Molecular Analysis of Patients with Neonatal-Onset UCDs

A definitive molecular diagnosis for every patient in our cohort is provided in Table 2. *In silico* analysis of the novel missense variants was carried out using PolyPhen-2 and SIFT.

**Figure 1:**
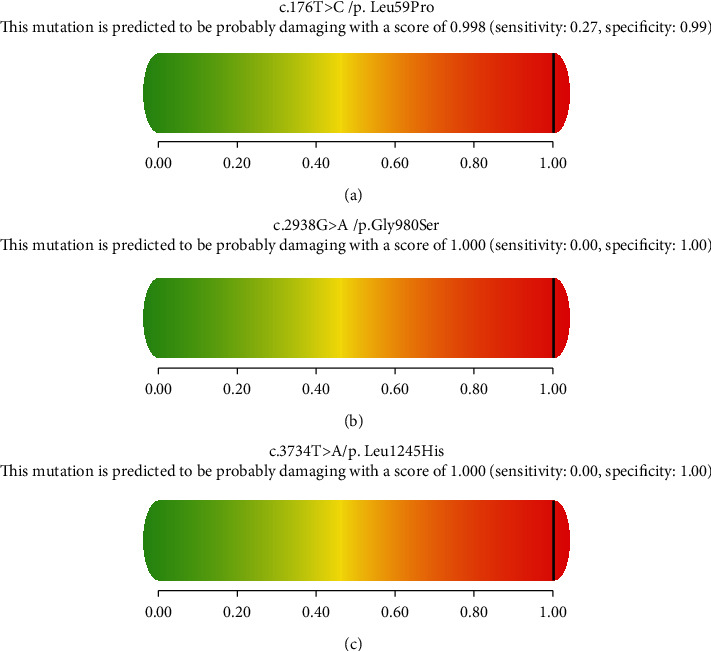
Pathogenicity analyses of (a) c.176T>C (p.L59P), (b) c.2938G>A (p.G980S), and (c) c.3734T>A (p.L1245H) by PolyPhen-2.

**Figure 2:**
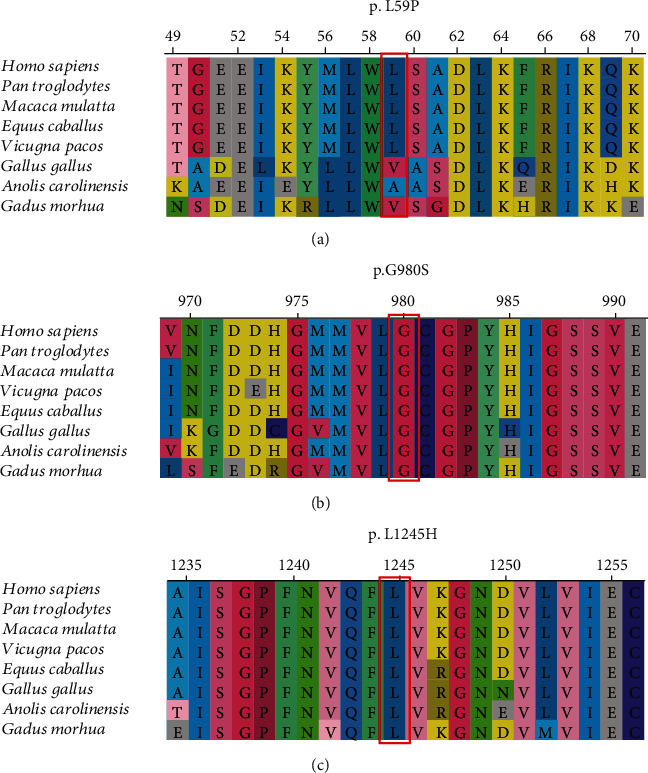
In silico analysis of (a) p.L59P, (b) p.G980S, and (c) p. L1245H in different species by ClustalX.

**Figure 3:**
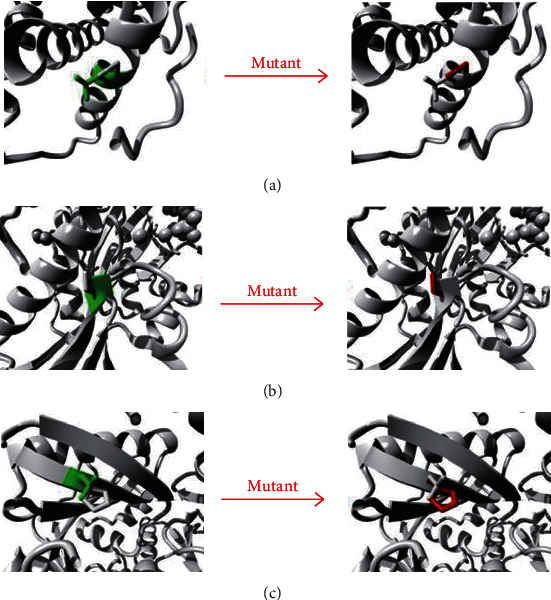
3D structure of wild type and mutant type of (a) p.L59P in OTC, (b) p.G980S in CPS1, and (c) p.L1245H in CPS1.

A total of 7 male patients (P1, P2, P3, P4, P5, P6, and P9) belonging to 6 families were diagnosed with OTCD. Molecular analysis revealed 6 different hemizygotes, all of which were inherited from their mother (Table 2). The reported variant, c.626C>T (p.A209V), was identified in two siblings (P5 and P6). The novel variant, c.176T>C (p.L59P), occurred with an amino acid change from a nonpolar amino acid of leucine (L) to another nonpolar amino acid of proline (P). It was found in a male (P3) presenting with irritability, seizure, and coma at the age of 2 days after birth, and the same one was documented in his mother. It was predicted to be “tolerated” by SIFT and “probably damaging” by PolyPhen-2 (Figure 1(a)). The conservation analysis showed that the wild-type residue—Leu—is not conserved at this position (Figure 2(a)). In addition, the OTC protein was built based on a homologous structure using HOPE. The structure (Figure 3(a)) revealed that the mutated residue—pro—disrupted an *α*-helix [23], at the N-terminal end of which the Ser-Thr-Arg-Thr-Arg motif is located and thereby might affect the function of the protein. Since the typical phenotypes of this patient appropriately matched the disorder, this novel variant was considered to be disease-causing. For the other four disease-causing mutations, we found that two variants, c.119G>A (p.R40H) (P1) and c.803T>C (p.M268T) (P4), which were reported to associate with late-onset presentations allied to neonatal-onset patients in this study, while c.583G>A (p.G195R) (P9) and c.540G>C (p.Q180H) were carried by two males with severe neonatal presentations, consistent with that reported in a previous study.

In this study, our NGS assays reported two patients (P7 and P8) carrying two compound heterozygous variants of c.2162G>A (p.R721Q)/c.2938G>A (p.G980S) and c.3734T>A (p.L1245H)/c.3784C>T (p.R1262∗) in the *CPS1* gene, individually. Two missense variants (c.2938G>A, p.G980S and c.3734T>A, p.L1245H) were novel. We analyzed their pathogenicity. Both of them were predicted to be “damaging” and “probably damaging” by SIFT and PolyPhen-2, respectively (Figures 1(b) and 1(c)). The conservation analyses showed that both sites were highly conservative in different species (Figures 2(b) and 2(c)). Furthermore, the CPS1 protein crystallographic structure of both variants revealed that the mutant residues might disturb the core structure of the carbamoylphosphate synthase domain, leading to the abolishment of the protein function (Figures 3(b) and 3(c)).

### 3.4. 180-Day Outcomes in Patients with Neonatal-Onset UCDs

During a 180-day follow-up interval, four patients (P1, P2, P4, and P8) in our cohort survived the neonatal period. At the end of the 6 months of age, 2 of the initially surviving patients (P1 and P2) died (Table 1).

#### 3.4.1. Survival and Coma at Admission

There were 4 episodes (P1, the first episode in P2, P7, and P8) of hyperammonemia without coma at the time of admission, and coma was also absent at the time of discharge in majority of them (P1, P2, and P7) (75.00%, 3/4). But three of them (P1, P2, and P8) died before 6 months of age (75.00%, 3/4). However, we found that the patients (the second episode in P2, P3, P4, P5, P6, and P9) were comatose at admission and no coma was present at the time of discharge in only one of them (P4). Five (P2, P3, P5, P6, and P9) died (83.33%, 5/6), all diagnosed with OTCD (Tables 1 and 2. Our results indicated that there were no significant differences in the survival rate for hyperammonemic episodes among neonates who were comatose at admission or not (*P* = 1.00).

#### 3.4.2. Survival and Severe Manifestation/Complication of Acute Hyperammonemia

Among 7 patients (P1, P2, P3, P5, P6, P8, and P9) who died, the following severe manifestations/complications of acute hyperammonemia were common, including seizures in 6 patients (P1, P2, P3, P4, P5, and P6), respiratory distress or failure in 5 (P3, P4, P5, P6, and P9), fever in 2 (P2 and P3), liver damage in 2 (P1 and P8), hyperventilation in 1 (P8), and cerebral hemorrhage in 1 patient (P3) individually (Table 1).

#### 3.4.3. Survival and Peak Ammonium Level

In our cohort, we found that four out of five patients (P3, P4, P5, P8, and P9) died, whose peak ammonium level was greater than 500 *μ*mol/L at presentation (survival 20%, 1/5), whereas the survival was not improved for those who (P1, P2, P6, and P7) had hyperammonemic episodes with a peak plasma ammonium level of less than 500 *μ*mol/L (survival 25%, 1/4).

#### 3.4.4. Survival and Treatment

Eight patients (P1, P3, P4, P5, P6, P7, P8, and P9) were initiated with protein restriction as soon as the onset of the first known hyperammonemia episode occurred, and only one patient (P2) continued protein intake till his second hyperammonemia episode. Six patients (P1, P2, P4, P7, P8, and P9) were treated with intravenous arginine infusion (100–150 mg/kg/day). The provision of adequate calorie was provided for all patients.

Six males (6/7, 85.71%) with OTCD (P1, P2, P3, P5, P6, and P9) died because of hyperammonemic crisis in the neonatal period or after. Among them, three preterm patients (P5, P6, and P9) did not survive the first hyperammonemic episode and died in their first week of life, whereas the others (P1, P2, and P3) died at an older age. Among patients who died, median ammonium levels were similar at baseline (a baseline ammonium level: the last value recorded before treatment) (653.32 ± 464.04 *μ*mol/L) and at the final assessment after treatment was initiated (599.98 ± 519.46 *μ*mol/L) (*P* = 0.248).

A full-term OTC-deficient male (P4) survived all known episodes in our follow-up interval. He presented with serious hyperammonemic encephalopathy at the age of 6 days. His ammonium level remained above 1000 *μ*mol/L within 8 hours after administration of the loading arginine infusion. Then, he was treated with a combination of a continuous renal replacement therapy for one day and peritoneal dialysis for 2 days. His plasma ammonium level decreased to normal, and he improved clinically. After discharge, he remained on protein restriction and oral sodium benzoate.

A CPS1-deficient female (P7) was in clinical remission until now. She presented with a sepsis and liver damage besides a high ammonium level at admission. She was provided with a combination of oral sodium benzoate (250 mg/kg/day) and intravenous arginine infusion (150 mg/kg/day) as an alternative pathway therapy immediately after her admission, resulting in a near-normal ammonium level and clinical improvement.

## 4. Discussion

In this study, we presented a detailed clinical course and genetic analysis of nine neonatal Chinese UCD cases. And our results showed that despite early diagnosis and treatment [24, 25], the 6-month survival of the severe early-onset type was not high.

Nine patients developed various symptoms within the first 7 days of life. The majority of this cohort (88.89%, 8/9) (P1, P2, P3, P4, P5, P6, P7, and P8) appeared to be healthy at birth except a premature male with OTCD (P9) who manifested tachypnea immediately after birth, whose mother had severe hyperammonemia during pregnancy. Acute encephalopathy and feeding problems (poor feeding in P1, P2, P3, P4, P6, P7, and P8 and no enteral feeding after birth in P5 and P9) were observed in all our patients, followed by breathing problems at the time of hospitalization. In the present study, phenotypic homogeneity of the same mutation was observed. The two male siblings (P5 and P6) with the same mutation in the *OTC* gene presented similarly except the exact onset time of their first hyperammonemia episode. However, our cohort also revealed phenotypic heterogeneity within the same family: an OTC-deficient male (P9) was characterized by a hyperammonemia crisis after birth and died at the third day of age; his heterozygous mother developed vomiting and altered consciousness during pregnancy, and successful management of her prenatal and postpartum blood ammonia level was achieved after the administration of pharmacologic nitrogen scavengers and protein limitation; his grandmother, heterozygous for the same mutation remained asymptomatic until then. Musalkova et al. [26] advocated that the heterogeneity of a group of female OTC carriers might be due to variable X-chromosome inactivation and environmental variables such as causes of catabolic stress as well.

Once a UCD is suspected, the diagnosis should be confirmed rapidly by a specialized metabolic laboratory, such as blood ammonia, blood amino acids, and urine organic acids. In this study, hyperammonemia of different levels was observed in all patients before the first week after birth. However, the concurrent low plasma citrulline was found only in three OTCD patients (P4, P5, and P6). The low level of urinary orotic acid was observed in one CPS1D patient (P7). So we cannot distinguish from different types of UCD due to similar intermediary metabolites or normal levels of some amino acids by mass spectrometry.

Molecular genetic testing is the primary method of diagnostic confirmation for all UCDs. As the use of NGS becomes standard practice, we expect that disease-causing mutations will be rapidly found in neonates who have severe hyperammonemia. It is reported that conventional Sanger sequencing cannot detect variants in about ~20% of OTCD patients [27, 28] and NGS had the advantage in detecting deletions spanning one or more exons rapidly [29]. Our study assessed one unreported variant in the *OTC* gene: c.176T>C (p.L59P), and two in the *CPS1* gene: c.2938G>A (p.G980S) and c.3734T>A (p.L1245H). Our results highlighted the relevance of combining molecular and bioinformatics analyses for accurate diagnosis and outcome prediction in patients with suspected UCDs. Additionally, our results prompted that family screening and genetic counseling by NGS-based tests can also be an important aspect of UCD prevention. There were three positive family histories derived from out dataset. The two female relatives of a family with male OTCD proband (P9) exhibited different phenotypes with the same mutation. The grandmother was apparently normal, while the mother presented severe hyperammonemia during pregnancy. Our finding supported that heterozygote detection of at-risk female relatives to determine their genetic status is most informative if the pathogenic variant has been identified in the proband. However, McCullough et al. reported that approximately 15% of female carriers become symptomatic, resulting to a diagnostic and ethical dilemma in screening of female OTC carriers [30]. Another family history told us that two male siblings (P5 and P6) were both having neonatal-onset OTCD. Since it is likely that subsequently affected males will have a similar presentation in a family with a history of neonatal-onset disease, prenatal genetic testing for a pregnancy at increased risk and preimplantation genetic diagnosis for OTC deficiency are necessary.

Finally, our results reflected the poor prognosis for neonatal-onset UCDs in China, compared with other populations [7]. To prevent metabolic emergencies and reduce high mortality, early diagnosis and immediate start of metabolic treatment in even asymptomatic patients are thought to be efficient ways to improve the outcome in a public health view. Current newborn screening (NBS) in China [31] cannot detect abnormal concentrations of analyses associated with UCDs. The introduction of expanded NBS by MS/MS was only carried out in some areas like Shanghai. However, none of the nine patients was identified due to the expanded NBS or the ordinary one. This notion was supported by the statement made by Kölker et al. [32] that some patients with early-onset OTCD might have been missed because of fatal neonatal crisis or might have died before screening results were available. So expanding neonatal screening for UCDs and shortening the turnout time are imperative in improving the sensitivity and specificity for screening these disorders.

## 5. Conclusion

In this study, we presented the detailed clinical features and genetic analysis of nine patients with neonatal-onset UCDs with poor 180-day-age outcomes and discovered three novel pathogenic variants in one OTCD and two CPS1D by NGS. Our results demonstrated that although biochemical phenotypes during the neonatal period were better than clinical profiles to predict OTCD, a molecular analysis was the gold standard to confirm the diagnosis, indicating that it is indispensable to expand NBS by MS/MS for some UCDs.

## Figures and Tables

**Table 1:** Clinical and laboratory data of the nine patients with neonatal-onset UCDs.

Patients	P1	P2	P3	P4	P5	P6	P7	P8	P9
Gender	M	M	M	M	M	M	F	M	M
Age at onset (days)	7	7	2	6	2	3	5	1	1
Age deceased (days)	170	165	7	Survival	3	7	Survival	5	3
Gestation age (weeks)	40.29	38.29	39.86	38.14	36.14	36.14	37.57	41	32
Birth weight (g)	4300	3900	3870	3750	2370	2230	3700	4080	1580
Complications during pregnancy	Uneventful	Uneventful	Uneventful	Uneventful	Uneventful	Uneventful	Uneventful	Uneventful	Developed acute hyperammonemia during pregnancy
Family history	Positive	—	—	—	Positive	Positive	—	—	Positive
Clinical course	Fulminant	Fulminant	Fulminant	Fulminant	Fulminant	Fulminant	Fulminant	Fulminant	Fulminant
Clinical features									
Seizures (HP:0001250)	Positive	Positive	Positive	Positive	Positive	Positive	—	—	—
Somnolence (HP:0001262)	Positive	Positive	Positive	Positive	Positive	Positive	Positive	Positive	Positive
Decreased liver function (HP:0001410)	Positive	—	—	—	—	—	Positive	—	—
Acute encephalopathy (HP:0006846)	Positive	Positive	Positive	Positive	Positive	Positive	Positive	Positive	Positive
Coma (HP:0001259)	—	No coma at the first episode, coma at the second episode	Positive	Positive	Positive	Positive	—	—	Positive
Fever (HP:0001945)	—	Positive	Positive	—	—	—	Positive	—	—
Hypothermia (HP:0002045)	—	—	—	—	Positive	Positive	—	Positive	Positive
Cyanosis (HP:0000961)	—	—	—	—	Positive	—	—	—	—
Neonatal breathing deregulation (HP:0002790)	—	—	Weak, need ventilation	Apnea, need ventilation	Apnea, need ventilation	Weak	—	Tachypnea	Apnea, need ventilation
Internal hemorrhage (HP:0011029)	—	—	Cerebral	—	—	—	—	—	—
Feeding difficulties (HP:0011968)	Positive	Positive	Positive	Positive	—	Positive	Positive	Positive	No enteral feeding after birth
Abdominal distention (HP:0003270)	—	—	—	—	—	—	Positive	—	—
Arterial blood gas analysis									
pH (reference, 7.25-7.45)	7.38	7.42	7.4	7.43	7.45	7.45	7.41	7.47	7.21
BE (reference, –3 to +3 mmol/L)	-1.8	-4.8	0.1	-1.3	-11.4	-5.9	-2.1	3.8	-10.1
Blood biochemical tests									
ALT (reference, 0-38 U/L)	125↑	34	25	25	23	5	107↑	45↑	21
Lactic acid (reference, 0.7-2.1 mmol/L)	1.9	6.4	8.2	3	7.3	7	1.5	2.4	8
Glucose (reference, 3.3-6.1 mmol/L)	4.2	5.7	2.4	3.5	1.1	3	4.8	3.8	2.9
Culture	—	—	—	—	—	—	Staphylococcus haemolyticus	—	—
Ammonia (reference, 9-30 *μ*mol/L)									
Peak pretreatment ammonia level	310↑	First episode: 126↑; second episode: 290↑	1367↑	1030↑	704.3↑	228.6↑	311↑	1004↑	1020↑
Final assessment after treatment	61↑	First episode: N/A; second episode:219↑	1100↑	23	1258.4↑	450.2↑	169↑	885↑	1100↑
Blood mass spectrometry profile									
Citrulline (reference, 5-40 *μ*mol/L)	5.953	First episode: N/A; second episode: 6.112	6.261	2.71↓	3.15↓	3.04↓	8.776	1.58↓	27.43
Alanine (reference, 70-350 *μ*mol/L)	213.014	First episode: N/A; second episode: 362.021↑	375.751↑	368.22↑	411.70↑	301.6	295.904	462.33↑	228.41
Glutamate (reference, 45-200 *μ*mol/L)	130.149	First episode: N/A; second episode: 213.893↑	404.649↑	652.67↑	265.41↑	195.73	263.168↑	185.21	145.66
Glutamine (reference, 2-35 *μ*mol/L)	14.047	First episode: N/A; second episode: 58.012↑	97.444↑	1577.35↑	156.6↑	100.62↑	14.751↑	1104.41↑	61.602↑
Ornithine (reference, 20-160 *μ*mol/L)	36.996	First episode: N/A; second episode: 100.351	122.83	38.5	29.17	31.98	41.037	54.43	161.66↑
Arginine (reference, 3-50 *μ*mol/L)	7.606	First episode: N/A; second episode: 21.207	91.894↑	6.12	20.14	9.07	20.12	5.26	97.43↑
Urinary organic acids									
Urinary orotic acid (reference, 0-2 mmol/L)	Absent	First episode: N/A; second episode: 357.21↑	641.56↑	352.13↑	80.5↑	12.5↑	0.15	0.41	1372.66↑
Urinary uracil (reference, 0-8 mmol/L)	4.76	First episode: N/A; second episode: 35.12↑	46.34↑	32.03↑	Absent	16.3↑	0.24	Absent	67.11↑
Chest X-ray									
Pneumonia	—	—	Positive	—	Positive	—	—	—	—
Pneumorrhagia	—	—	—	—	—	—	—	—	—
Echocardiography	N/A	N/A	N/A		N/A	N/A			N/A
Ejection fraction				62%			59%	64%	
Patent ductus arteriosus				Positive			—	—	
Decision making	—	—	Give up	—	—	Give up	—	Give up	Give up

↑: elevated, ↓: decreased. N/A: not mentioned.

**Table 2:** Molecular profiles of 9 neonatal-onset UCD patients.

Patient number	Molecular diagnostic technology	Family member tests	Gene	Nucleotide aberration	Amino acid change	Molecular diagnosis	Inheritance pattern	Zygosity	Parent of origin
P1	TES panel	Trio+1 brother	OTC (NM_000531.6)	c.119G>A	p.R40H	Ornithine transcarbamylase deficiency (MIM:311250)	XR	Hemi	Inherited/mother
P2	TES panel	Trio	OTC (NM_000531.6)	c.540G>C	p.Q180H	Ornithine transcarbamylase deficiency (MIM:311250)	XR	Hemi	Inherited/mother
P3	TES panel	Trio	OTC (NM_000531.6)	*c.176T>C*	p.L59P	Ornithine transcarbamylase deficiency (MIM:311250)	XR	Hemi	Inherited/mother
P4	WES	Trio	OTC (NM_000531.6)	c.803T>C	p.M268T	Ornithine transcarbamylase deficiency (MIM:311250)	XR	Hemi	Inherited/mother
P5	WES	Trio+1 sib	OTC (NM_000531.5)	c.626C>T	p.A209V	Ornithine transcarbamylase deficiency (MIM:311250)	XR	Hemi	Inherited/mother
P6	WES	Trio+1 sib	OTC (NM_000531.5)	c.626C>T	p.A209V	Ornithine transcarbamylase deficiency (MIM:311250)	XR	Hemi	Inherited/mother
P7	WES	Trio	CPS1 (NM_001875.4)	c.2162G>A; *c.2938G>A*	p.R721Q; p.G980S	Carbamoylphosphate synthetase I deficiency (MIM:237300)	AR	Het	Inherited/father+mother
P8	WES	Trio	CPS1 (NM_001875.5)	c.3784C>T; *c.3734T>A*	p.R1262∗; p.L1245H	Carbamoylphosphate synthetase I deficiency (MIM:237300)	AR	Het	Inherited/father+mother
P9	TES panel	Trio	OTC (NM_000531.5)	c.583G>A	p.G195R	Ornithine transcarbamylase deficiency (MIM:311250)	XR	Hemi	Inherited/mother

AR: autosomal recessive inheritance disease; XR: X-linked recessive inheritance disease; Het: heterozygous; Hemi: hemizygous; hom: homozygous; TES: targeted exome sequencing; WES: whole-exome sequencing. Italicized variants were unreported previously.

## Data Availability

The data used to support the findings of this study are included within the article.

## Supplementary Materials

Supplementary Materials Supplementary Methods: a section about the list of a specific neonatal metabolism disease 175-gene panel (designed by MyGenostics, Beijing, China) for 4 patients in our cohort.

## References

[1] Häberle J., Burlina A., Chakrapani A. (2019). Suggested guidelines for the diagnosis and management of urea cycle disorders: first revision. *Journal of Inherited Metabolic Disease*.

[2] Díez-Fernández C., Gallego J., Häberle J., Cervera J., Rubio V. (2015). The study of carbamoyl phosphate synthetase 1 deficiency sheds light on the mechanism for switching on/off the urea cycle. *Journal of Genetics and Genomics*.

[3] Caldovic L., Abdikarim I., Narain S., Tuchman M., Morizono H. (2015). Genotype–phenotype correlations in ornithine Transcarbamylase deficiency: a mutation update. *Journal of Genetics and Genomics*.

[4] Sarker S. K., Islam M. T., Biswas A. (2019). Age-specific cut-off values of amino acids and acylcarnitines for diagnosis of inborn errors of metabolism using liquid chromatography tandem mass spectrometry. *BioMed Research International*.

[5] Kwon J. M. (2018). Testing for inborn errors of metabolism. *Continuum*.

[6] Kapil S., Fishler K. P., Euteneuer J. C., Brunelli L. (2019). Many newborns in level IV NICUs are eligible for rapid DNA sequencing. *American Journal of Medical Genetics. Part A*.

[7] Batshaw M. L., Tuchman M., Summar M., Seminara J., Members of the Urea Cycle Disorders Consortium (2014). A longitudinal study of urea cycle disorders. *Molecular Genetics and Metabolism*.

[8] Maestri N. E., Clissold D., Brusilow S. W. (1999). Neonatal onset ornithine transcarbamylase deficiency: a retrospective analysis. *The Journal of Pediatrics*.

[9] Zheng Z., Lin Y., Lin W. (2020). Clinical and genetic analysis of five Chinese patients with urea cycle disorders. *Molecular Genetics & Genomic Medicine*.

[10] Shao Y., Jiang M., Lin Y. (2017). Clinical and mutation analysis of 24 Chinese patients with ornithine transcarbamylase deficiency. *Clinical Genetics*.

[11] Gong Z. W., Han L. S., Ye J. (2016). Applying multiplex ligation-dependent probe amplification in the diagnosis of 5 cases with ornithine transcarbamylase deficiency. *Chinese Journal of Pediatrics*.

[12] Köhler S., Vasilevsky N. A., Engelstad M. (2017). The human phenotype ontology in 2017. *Nucleic Acids Research*.

[13] Richards S., Aziz N., Bale S. (2015). Standards and guidelines for the interpretation of sequence variants: a joint consensus recommendation of the American College of Medical Genetics and Genomics and the Association for Molecular Pathology. *Genetics in Medicine*.

[14] Brandt T., Sack L. M., Arjona D. (2020). Adapting ACMG/AMP sequence variant classification guidelines for single-gene copy number variants. *Genetics in Medicine*.

[15] Stenson P. D., Mort M., Ball E. V. (2017). The Human Gene Mutation Database: towards a comprehensive repository of inherited mutation data for medical research, genetic diagnosis and next-generation sequencing studies. *Human Genetics*.

[16] Lek M., Karczewski K. J., Minikel E. V. (2016). Analysis of protein-coding genetic variation in 60,706 humans. *Nature*.

[17] Adzhubei I. A., Schmidt S., Peshkin L. (2010). A method and server for predicting damaging missense mutations. *Nature Methods*.

[18] Kumar P., Henikoff S., Ng P. C. (2009). Predicting the effects of coding non-synonymous variants on protein function using the SIFT algorithm. *Nature Protocols*.

[19] Larkin M. A., Blackshields G., Brown N. P. (2007). Clustal W and Clustal X version 2.0. *Bioinformatics*.

[20] Venselaar H., Te Beek T. A., Kuipers R. K., Hekkelman M. L., Vriend G. (2010). Protein structure analysis of mutations causing inheritable diseases. An e-Science approach with life scientist friendly interfaces. *BMC Bioinformatics*.

[21] Hehir-Kwa J. Y., Pfundt R., Veltman J. A. (2015). Exome sequencing and whole genome sequencing for the detection of copy number variation. *Expert Review of Molecular Diagnostics*.

[22] Macklin S. K., Jackson J. L., Atwal P. S., Hines S. L. (2019). Physician interpretation of variants of uncertain significance. *Familial Cancer*.

[23] Shi D., Morizono H., Yu X., Tong L., Allewell N. M., Tuchman M. (2001). Human ornithine transcarbamylase: crystallographic insights into substrate recognition and conformational changes. *The Biochemical Journal*.

[24] Yamaguchi S., Brailey L. L., Morizono H., Bale A. E., Tuchman M. (2006). Mutations and polymorphisms in the human ornithine transcarbamylase (OTC) gene. *Human Mutation*.

[25] Matsumoto S., Häberle J., Kido J., Mitsubuchi H., Endo F., Nakamura K. (2019). Urea cycle disorders-update. *Journal of Human Genetics*.

[26] Musalkova D., Sticova E., Reboun M. (2018). Variable X-chromosome inactivation and enlargement of pericentral glutamine synthetase zones in the liver of heterozygous females with OTC deficiency. *Virchows Archiv*.

[27] Yokoi K., Nakajima Y., Inagaki H., Tsutsumi M., Ito T., Kurahashi H. (2018). Exonic duplication of the OTC gene by a complex rearrangement that likely occurred via a replication-based mechanism: a case report. *BMC Medical Genetics*.

[28] Shchelochkov O. A., Li F.-Y., Geraghty M. T. (2009). High-frequency detection of deletions and variable rearrangements at the ornithine transcarbamylase (OTC) locus by oligonucleotide array CGH. *Molecular Genetics and Metabolism*.

[29] Yao R., Zhang C., Yu T. (2017). Evaluation of three read-depth based CNV detection tools using whole-exome sequencing data. *Molecular Cytogenetics*.

[30] McCullough B. A., Yudkoff M., Batshaw M. L., Wilson J. M., Raper S. E., Tuchman M. (2000). Genotype spectrum of ornithine transcarbamylase deficiency: correlation with the clinical and biochemical phenotype. *American Journal of Medical Genetics*.

[31] Therrell B. L., Padilla C. D. (2018). Newborn screening in the developing countries. *Current Opinion in Pediatrics*.

[32] Kölker S., Cazorla A. G., Valayannopoulos V. (2018). The phenotypic spectrum of organic acidurias and urea cycle disorders. Part 1: the initial presentation. *Journal of Inherited Metabolic Disease*.

