# Supplementary material for: The Application of Next-Generation Sequencing (NGS) in Neonatal-Onset Urea Cycle Disorders (UCDs): Clinical Course, Metabolomic Profiling, and Genetic Findings in Nine Chinese Hyperammonemia Patients
Source: Biomed Res Int. 2020 Aug 31;2020:5690915. doi: 10.1155/2020/5690915 (PMC7479453; doi:10.1155/2020/5690915)
Supplement: Supplementary Materials — Supplementary Methods: a section about the list of a specific neonatal metabolism disease 175-gene panel (designed by MyGenostics, Beijing, China) for 4 patients in our cohort. [file 5690915.f1.docx]

**Supplementary Materials**

Supplementary Methods: a section about the list of a specific neonatal metabolism disease 175-gene panel (designed by MyGenostics, Beijing, China) for 4 patients in our cohort.

**List of all covered genes in the Neonatal metabolism panel**

| Name for TES Panels | Genes involved in the panel | | | | | | | | | |
| --- | --- | --- | --- | --- | --- | --- | --- | --- | --- | --- |
| Neonatal metabolism panel |  | | | | | | | | | |
|  | *PAH* | *L2HGDH* | *DHTKD1* | *CTH* | *ARX* | *ATP7B* | *GCSH* | *PTS* | *D2HGDH* | *GALK1* |
|  | *MTHFR* | *SLC6A8* | *MMAA* | *G6PD* | *CPS1* | *GCH1* | *IDH2* | *INPP5E* | *MTRR* | *GAMT* |
|  | *MMAB* | *ATP7A* | *OTC* | *QDPR* | *ETFA* | *LAMP2* | *MTR* | *GATM* | *ABCD4* | *PTPN11* |
|  | *ASS1* | *PCBD1* | *ETFB* | *MAOA* | *GNMT* | *ERCC8* | *GPHN* | *MVK* | *ASL* | *SPR* |
|  | *ETFDH* | *PNPLA2* | *AHCY* | *ERCC6* | *MCEE* | *ACSF3* | *ARG1* | *FAH* | *BCAT1* | *SLC2A1* |
|  | *SLC25A13* | *OGDH* | *OAT* | *TAT* | *BCAT2* | *HSD17B10* | *HCFC1* | *FH* | *NAGS* | *HPD* |
|  | *SLC22A5* | *SLC2A2* | *GLUD1* | *FOLR1* | *LMBRD1* | *AASS* | *SLC7A7* | *HGD* | *CPT1A* | *SLC3A1* |
|  | *GLUL* | *FOLR2* | *GCDH* | *ABHD5* | *MAT1A* | *HAL* | *CPT2* | *SLC7A9* | *BCKDHA* | *DHFR* |
|  | *HMGCL* | *ACAT1* | *CBS* | *UROC1* | *SLC25A20* | *BCKDHB* | *DDC* | *AUH* | *ADK* | *SUOX* |
|  | *FTCD* | *MLYCD* | *TYMP* | *DBT* | *PHGDH* | *TAZ* | *ALDH6A1* | *MOCS1* | *GLDC* | *ACADSB* |
|  | *TK2* | *DLD* | *PSAT1* | *OPA3* | *ASPA* | *MOCS2* | *AMT* | *ACADS* | *DGUOK* | *SARDH* |
|  | *ABAT* | *SERAC1* | *DBH* | *NR0B1* | *ACADM* | *POLG* | *PRODH* | *ALDH5A1* | *FBXL4* | *MCCC2* |
|  | *SOX9* | *ACADVL* | *SUCLA2* | *ALDH4A1* | *SRY* | *ALPL* | *KMT2D* | *PCCA* | *CYP21A2* | *HADHA* |
|  | *MPV17* | *SLC6A20* | *AR* | *PNPO* | *KDM6A* | *PCCB* | *CYP11B1* | *HADHB* | *C10orf2* | *SLC6A19* |
|  | *HSD17B3* | *ETHE1* | *SGSH* | *HLCS* | *HSD3B2* | *HADH* | *RRM2B* | *SLC36A2* | *SRD5A2* | *FOXG1* |
|  | *NAGLU* | *BTD* | *CYP17A1* | *ACAD8* | *SUCLG1* | *IVD* | *NR5A1* | *MECP2* | *HGSNAT* | *PC* |
|  | *StAR* | *TH* | *SLC25A4* | *MCCC1* | *WT1* | *CDKL5* | *GNS* | *MUT* | *ALDH7A1* | *GLYCTK* |
|  | *MMADHC* | *SLC46A1* | *SLC19A1* | *SLC25A15* | *MMADHC* |  |  |  |  |  |
